# Heterogeneity in clinical sequencing tests marketed for autism spectrum disorders

**DOI:** 10.1038/s41525-018-0066-3

**Published:** 2018-09-19

**Authors:** Ny Hoang, Janet A. Buchanan, Stephen W. Scherer

**Affiliations:** 1 Department of Genetic Counselling, The Hospital for Sick Children, Toronto, ON Canada; 2 Genetics and Genome Biology, The Hospital for Sick Children, Toronto, ON Canada; 3 Department of Molecular Genetics, University of Toronto, Toronto, ON Canada; 4 The Centre for Applied Genomics, The Hospital for Sick Children, Toronto, ON Canada; 5 McLaughlin Centre and Department of Molecular Genetics, Faculty of Medicine, University of Toronto, Toronto, ON Canada

Autism spectrum disorders (ASD) is now a high profile and common concern in the population. Diagnosis has long been based on clinical findings,^1^ but with increased recognition of a strong genetic contribution and a subset of cases with an underlying genetic syndrome,^2^ clinical laboratory testing to search for genomic risk variants is now an important component of the diagnostic work-up. Chromosomal microarray is currently the recommended first-tier genetic test for ASD,^3^ revealing deleted and duplicated segments of DNA. These relatively large copy number variations (CNVs), often affecting several genes, can account for 5–25% of ASD cases, depending on the cohort examined.^4^

The introduction of next-generation sequencing (NGS) now allows for a more in-depth look at ASD’s genetic landscape. Besides CNVs, genomic risk factors for ASD can include single-nucleotide variants, insertion/deletions, and complex structural variations, contained in potentially hundreds of different genes, which may be involved in complex interactions.^4–9^ The NGS technology may be targeted to a selection of genes of interest, or to the coding portions of all genes (exome sequencing), or for entire genome sequencing. Such sequencing approaches are now being marketed for use as second-tier tests for ASD, particularly when chromosomal microarray analysis has not revealed any explanation for the clinical presentation.

We undertook to survey the scope of sequencing tests for ASD (or including ASD) that are primarily being marketed by commercial laboratories as adjuncts or follow-up to chromosomal microarrays. Perhaps not surprisingly, because this is a new territory and guidelines are not yet developed, we found significant heterogeneity among such laboratories with respect to the tests they offer. The most striking finding was the variable number of genes being tested for on panels marketed for ASD (range, 11–2562), with little content overlap, albeit, these encompassed ASD-specific as well as much larger ASD-inclusive panels (Table 1).

**Table 1:** Clinical ASD gene sequencing panels available as of June 2018

Number	Laboratory company	Test name	Number of genes included	Gene list provided^a^	Gene selection criteria/rationale provided^b^	Certification/accreditation notes provided^b^
1	Ambry Genetics	AutismNext	48	●	●	●
2	ApolloGen	Autism Spectrum Disorders Panel Test	125	●		●
3	Asper Biogene Asper Biogene LLC	Autism Spectrum Disorders NGS panel	52	●		●
4	Ayass BioScience	AUTISM – Genetic Testing	98	○	●	●
5	Blueprint Genetics	Autism Spectrum Disorder Panel	26	●	●	●
6	CEN4GEN	Autism Spectrum Disorders: Gene Panel	63	●		
7	Center for Human Genetics, Inc	Autism Spectrum Disorders 53-Gene Panel	53	●	●	●
8	Centogene	Syndromic autism panel	50	●	●	●
9	CGC Genetics	Autism	28	●	●	●
10	EGL Genetic Diagnostics (Emory)	Autism Spectrum Disorders: Tier 2 Panel	63	●		●
11	Fulgent Genetics	Autism NGS Panel	106	●		●
12	GeneDx	Autism/ID Xpanded Panel	2562	●	●	●
13	GENETAQ	Autism	28	●		●
14	Greenwood Genetic Center Diagnostic Laboratories	Syndromic Autism Sequencing Panel	83	●	●	●
15	Munroe-Meyer Institute	Autism/Intellectual Disability/Multiple Anomalies Panel	117	●	●	●
16	Lineagen	NextStepDx PLUS	936	○	●	
17	Michigan Medical Genetics Laboratories	Autism / Intellectual Disability Panels (Tier 2 and Tier 3)	11	●	●	●
18	MNG Laboratories	Comprehensive Intellectual Disability/Autism	901	●	●	●
19	PreventionGenetics	Autism Spectrum Disorders and Intellectual Disability (ASD-ID) Comprehensive Sequencing Panel with CNV Detection **AND**	1908	●	●	●
		Autism Spectrum Disorders Sequencing Panel with CNV Detection	108			
20	Reference Laboratory Genetics	Autism Spectrum Disorders (Expanded Panel)	77	●		●
21	Sema4	Autism Spectrum Disorder Sequencing Panel	30	●		●

○ = provided upon request

^a^See Supplementary Table S1 for full gene list

^b^See Supplementary Table S5 for details

Our search began in 2017, using the former GeneTests and current Genetic Testing Registry as resources, supplemented by an internet search (terms: “autism panel”, “autism lab sequencing”, “autism genetic test”). To focus on gene panels, we excluded biochemical assays, chromosomal microarrays, and sequencing tests involving fewer than three genes. To focus on ASD, we excluded tests targeted for general neurodevelopmental disorders, seizure disorders, and intellectual disability, unless they specified autism. That search resulted in 20 DNA testing laboratories offering an ASD gene panel. Updating the survey in June 2018 (adding use of a new resource, Concert Genetics—www.concertgenetics.com), we found that four panels were no longer offered, but five were newly available, for an updated total of 21 laboratories (Table 1). We then went to the individual laboratory websites for further information.

We compared ASD gene lists from panels of the 21 laboratories. Each entire panel is listed in Supplementary Table S1. Supplementary Table S2 shows the 178 genes included in at least five lab panels (“shared genes”) in order of listing frequency. Table 2 summarizes the top 16 most commonly listed genes, along with their associated genetic disorders according to the Online Mendelian Inheritance in Man (OMIM).^10^ The latter shows that most (12/16) of these genes are associated with genetic syndromes, where the primary phenotype involves physical/systemic features and not ASD. Almost half of these genes (7/16) were located on the X chromosome. Only one gene was included on all panels offered by the 21 labs: *MECP2*, which is associated with Rett syndrome (previously considered part of the ASD spectrum, but no longer so under criteria of the *Diagnostic and statistical manual of mental disorders* (5th edn)).^1^ There were 63 genes shared among at least 10 lists, but the vast majority of the cumulative list of 2928 unique genes were included by fewer than five laboratories. There were, nonetheless, some pockets of significant overlap. Two pairs of laboratories each posted identical ASD gene lists. Two other laboratories had lists that encompassed those of separate labs, but with additional genes to create their own collection.

**Table 2:** Summary of findings from gene list comparison and list of most commonly listed genes

Key findings		
● Number of genes included on ASD gene sequencing panels ranged from 11 to 2562 ● 2928 unique genes identified across 21 labs ● When comparing the gene lists of 21 labs (see Supplementary Table S1) ○ Only one gene (*MECP2*) was shared by all labs ○ 63 genes shared by at least 10/21 labs ○ Two sets of labs had the same gene lists ● Of the top 16 genes shared by 16 or more panels, ○ 12 were associated with known OMIM syndromes^a^ primarily involving physical/systemic features, of which 3 (*MECP2*, *PTEN*, and *CNTNAP2*) included autism susceptibility ○ 4 (*NLGN4X*, *PTCHD1*, *NLGN3*, and *FOXP1*) were associated with primarily a neurocognitive presentation (ASD, intellectual disability) in OMIM ○ 2 (*UBE3A* and *SHANK3*) overlapped with known ASD-associated CNV regions ● The top 178 genes shared across 21 labs did NOT include 39 genes cited on 2 or more ASD gene lists^c^		
Top shared genes^b^		
**Gene name**	**Labs**	**OMIM gene association (OMIM#)** ^a^
*MECP2* ^c^	21/21	Encephalopathy (300673); Mental retardation (300260)(300055); Rett syndrome (312750); Autism susceptibility (300496)
*NLGN4X*	20/21	Mental retardation (300495); Asperger syndrome susceptibility (300497); Autism susceptibility (300495)
*CACNA1C* ^c^	19/21	Brugada syndrome 3 (611875); Timothy syndrome (601005)
*NRXN1* ^c^	19/21	Pitt-Hopkins-like syndrome 2 (614325); Schizophrenia susceptibility (614332)
*PCDH19*	19/21	Epileptic encephalopathy (300088)
*PTCHD1* ^c^	19/21	Autism susceptibility (300830)
*UBE3A*	19/21	Angelman syndrome (105830)
*NLGN3* ^c^	18/21	Asperger syndrome susceptibility (300494); Autism susceptibility (300425)
*PTEN* ^c^	18/21	Bannayan-Riley-Ruvalcaba syndrome (153480); Cowden syndrome 1/ Lhermitte-Duclos syndrome (158350); Macrocephaly/autism syndrome (605309); VATER association with macrocephaly and ventriculomegaly (276950); Glioma susceptibility (613028); Meningioma (607174); Prostate cancer (176807)
*SHANK3* ^c^	18/21	Phelan-McDermid syndrome (606232); Schizophrenia (613950)
*CDKL5* ^c^	17/21	Epileptic encephalopathy (300672)
*CNTNAP2*	17/21	Cortical dysplasia-focal epilepsy syndrome/ Pitt-Hopkins like syndrome 1 (610042); Autism susceptibility (612100)
*DHCR7* ^c^	17/21	Smith-Lemli-Opitz syndrome (270400)
*FOXP1* ^c^	17/21	Mental retardation with language impairment and with or without autistic features (613670)
*NSD1* ^c^	17/21	Leukemia (601626); Sotos syndrome 1 (117550)
*ARX* ^c^	16/21	Epileptic encephalopathy (308350); Hydranencephaly with abnormal genitalia (300215); Lissencephaly (300215); Mental retardation (300419); Partington syndrome (309510); Proud syndrome (300004)

^a^Online Mendelian Inheritance in Man (OMIM) is a continuously updated catalog of human genes and genetic disorders and traits, with particular focus on the molecular relationship between genetic variation and phenotypic expression. omim.org

^b^List of the top 16 genes shared by 16 or more clinical ASD sequencing panels

^c^Gene listed on 2 or more of the following: SFARI list^11^ with gene score of 1 or 2; SPARK list^12^ as of April 2017; MSSNG list published in 2017;^8^ ASC list^13^ of genes with *q* value <0.3

We then compared the gene lists ascertained here to four lists from academic research projects that identify genes with strong association to ASD: Simons Foundation Autism Risk Initiative (SFARI),^11^ Simons Foundation Powering Autism Research for Knowledge (SPARK),^12^ Autism Speaks – MSSNG,^8^ and Autism Sequencing Consortium (ASC)^13^ (Supplementary Table S2). Comparing these lists, we found 15 genes to be shared by all, and an additional 24 genes were common to 3 of the 4 lists (Supplementary Table S3). We noted that 39 ASD risk genes identified by at least two of these research sources were not included among the top 178 genes listed by the commercial labs (Supplementary Table S4). Moreover, seven well-studied genes (*ADNP*, *ARID1B*, *CHD8*, *POGZ*, *SCN2A*, *SLC6A1*, and *SYNGAP1*) identified to be important for ASD by the SFARI, SPARK, MSSNG, and ASC research projects, are not found among the commercial list of the top 47 shared genes (Supplementary Table S2).

How were these lists derived by the 21 clinical laboratories? We looked for such information from the websites, documented the mention of gene selection criteria or rationale (Table 1) and summarized the comments found (Supplementary Table S5). Some labs indicated nothing, some made general comments, some specified a filter of “syndromic autism”, and a few provided notes with or without literature citations. Favorably, one lab provided a dated record of updates to their panel.

From the respective websites, information about sequencing and analysis approaches used by the various laboratories were rarely obvious and sometimes unstated. A few of the labs undertook exome sequencing followed by selective analysis of an ASD gene panel. A few other labs stated that they applied NGS to “selected” or “targeted” genes only, and most did not provide any specification. Some added mention of exons or coding sequences; some also mentioned introns, splice junctions, or non-coding sequence. One lab clearly said they used Sanger sequencing only, and some noted that adjunct Sanger sequencing was applied either for regions poorly covered with NGS or for confirmation of significant findings before reporting.

Our ascertainment was intended to identify clinical tests; therefore, we searched for documentation of laboratory qualification and tabulated key findings (Table 1 and Supplementary Table S5). Additionally, a few websites specified that tests were not direct-to-consumer or could only be ordered by a health professional. Several labs mentioned that the test was intended for individuals with symptoms or clinical diagnosis of ASD, some limiting this to “syndromic”; few mentioned “pre-symptomatic”, “prenatal”, or “carrier” testing. Some described in-house genetic counseling support and/or the qualifications of those preparing reports. Few described their variant reporting protocol, some mentioning the classification scheme of the American College of Medical Genetics and Genomics.^14^

The diversity unearthed by this survey applied not only to the specific gene tests offered for ASD, but to the extent of supporting information provided by the companies who market these services. Consumers would benefit from greater transparency (including evidence) about items such as gene inclusion criteria, dates of updates, technologies used, interpretation of variants, reporting standards, ownership of data, secondary use policies, etc.

ASD is clearly a heterogeneous disorder, both in clinical presentation and in terms of the underlying etiology.^15^ In fact, the genetic predisposition for ASD may be different in almost every individual.^6,9^ Furthermore, the evidence for association between any given variant and ASD is wide-ranging, and even when an association is strongly supported, there is usually variable expressivity or some degree of non-penetrance reflected in the evidence.^12,15^ The NGS tests currently marketed for ASD are mostly being used in a confirmatory manner, in the context of clinical findings. However, expectations are rising, from families and their healthcare providers, to use the same DNA tests to enable early diagnosis, prognosis, and medical management of ASD, and to assess familial risk. This survey highlights the need to develop a clinically validated list of genes appropriate for clinical laboratory analysis for ASD to meet these growing demands.

## Electronic supplementary material

Supplementary Table S1

Supplementary Table S2

Supplementary Table S3

Supplementary Table S4

Supplementary Table S5

## Electronic supplementary material

**Supplementary information** accompanies the paper on the *npj Genomic Medicine* website (10.1038/s41525-018-0066-3).
